# Blood meal induced regulation of the chemosensory gene repertoire in the southern house mosquito

**DOI:** 10.1186/s12864-017-3779-2

**Published:** 2017-05-19

**Authors:** Tanvi Taparia, Rickard Ignell, Sharon Rose Hill

**Affiliations:** 1 Unit of Chemical Ecology, Department of Plant Protection Biology, Swedish University of Agricultural Sciences, Alnarp, Sweden; 2 Present Address: Department of Environmental Sciences, Wageningen University and Research, Wageningen, The Netherlands

**Keywords:** *Culex quinquefasciatus*, Chemoreceptors, Chemosensory proteins, Host seeking, Blood meal, Resting, Transcriptome, Olfaction

## Abstract

**Background:**

The southern house mosquito, *Culex quinquefasciatus*, is one of the most prevalent vectors of lymphatic filariasis and flavivirus-induced encephalitis. Its vectorial capacity is directly affected by its reproductive feeding behaviors, such as host seeking, blood feeding, resting, and egg laying. In mosquitoes, these gonotrophic behaviors are odor-mediated and regulated following blood feeding. Immediately after a blood meal, female mosquitoes show reduced olfactory responsiveness and flight activity, as they enter a resting state. Insights into antennal chemosensory gene regulation at this time period can provide a foundation to identify targets involved in the state switch between host seeking and resting.

**Results:**

This study used quantitative gene expression analyses to explore blood meal induced regulation of chemosensory gene families in the antennae of 6 days post-emergence *C. quinquefasciatus* females. Improved annotations for multiple chemosensory gene families, and a quantitative differential gene expression analysis between host seeking and 24 h post- blood fed females of the same age, allowed for the detection of transcripts that potentially play a role in the switch from host seeking to resting, in *C. quinquefasciatus*. The expression profiles of chemosensory genes varied significantly between the two treatments.

**Conclusions:**

Annotations for chemosensory gene repertoires in *C. quinquefasciatus* have been manually curated and corrected for 3’ exon choice and transcript length, through sequence and transcriptome analyses. The gene expression analyses identified various molecular components of the peripheral olfactory system in *C. quinquefasciatus*, including odorant receptors, ionotropic receptors, odorant binding proteins and chemosensory proteins, that are regulated in response to blood feeding, and could be critical for the behavioral switch from host seeking to resting. Functional characterization of these proteins in the future can identify targets essential for the females’ gonotrophic behaviors, and can be used to design novel vector control strategies.

**Electronic supplementary material:**

The online version of this article (doi:10.1186/s12864-017-3779-2) contains supplementary material, which is available to authorized users.

## Background

Females of the southern house mosquito, *Culex quinquefasciatus,* require a blood meal to complete each gonotrophic cycle, comprised of alternately host-seeking, blood feeding, resting, and egg laying [1]. This successive reproductive feeding behavior puts more than one fifth of the world’s population at risk of vector borne diseases such as lymphatic filariasis and flavivirus-induced encephalitis, including West Nile fever [2]. Similar to other mosquito species, *C. quinquefasci* *atus* relies heavily on olfaction to mediate gonotrophic behaviors [3, 4]. Characterization of the gene expression that underpins the switching between the stereotypic odor-mediated behaviors may identify targets that can be used for the development of novel monitoring and control strategies.

Behavioral studies indicate that *C. quinquefasci* *atus* are attracted to birds, and bird-derived odorants, as well as human emanates during host seeking [5, 6]. The identity of these volatiles is only now being described, with the sole known attractant to date being nonanal [7]. In addition, some volatiles driving the oviposition behavior of *C. quinquefasci* *atus*, ca. 72 hours post-blood meal, have been identified from the preferred egg laying substrate, fermenting vegetation, including for example 4-methylphenol, 3-methylindole, indole, and nonanal [8]. Physiological recordings from the sensilla on the main olfactory organ, the antenna, have shown that olfactory receptor neurons (ORNs) of *C. quinquefasci* *atus* and other mosquitoes are tuned to these as well as other odors present in host and oviposition site emissions [9–14].

In insects, odors are detected by the molecular components of the peripheral olfactory system [15]. The lymph soluble proteins, odorant binding proteins (OBPs) and chemosensory proteins (CSPs), are believed to be involved in salient odorant recognition and translocation of volatile chemicals to the ORN dendritic membrane. At the membrane, odorant receptors (ORs), ionotropic receptors (IRs), gustatory receptors (GRs), and sensory neuron membrane proteins (SNMPs) are involved in odorant recognition and transduction of volatiles into electrical signals [16]. Finally, odorant degrading enzymes (ODEs) have been proposed to mediate the subsequent volatile clearance from the lymph [16]. While the majority of members from each of these gene families have been identified in the yellow fever mosquito, *Aedes aegypti* [17], and the malaria mosquito, *Anopheles gambiae* [18, 19], the annotation of these genes is ongoing in *C. quinquefasci* *atus* [20–22]. The genome-wide duplication events in *C. quinquefasci* *atus* [21], coupled with the rapid expansion of the chemosensory gene families in insects [15, 23], has hitherto constrained the annotation process.

Transcriptome analyses across the antennae of *Ae. aegypti* and *An. gambiae* reveal a transient change in expression of chemosensory genes during the first gonotrophic cycle, evident already 24 h post-blood meal [17, 24, 25]. In general, an overall reduction in chemosensory gene transcripts in the antennae of both *Ae. aegypti* and *An. gambiae* is observed following a blood meal, with only a few olfactory receptors being significantly up-regulated [17, 24]. A comparison between *Ae. aegypti* and *An. gambiae* indicates that the regulation of chemoreceptor expression post-blood meal is species specific among putative homologues [17, 24]. Moreover, of the five OBPs up-regulated 24 h post-blood meal (>3 fold) in *An. gambiae*, three show species specific regulation, while the up-regulation of *AgOBP9* and *AgOBP54* is reflected in their *Ae. aegypti* putative homologues [17, 24]. The observed regulation of select chemosensory genes following a blood meal coincides with the olfactory refractoriness in the host-seeking behavior [26–28], reduced flight activity [29], and the change in electrophysiological responses of olfactory sensilla to volatiles [10, 13], in recently blood fed mosquitoes. This endogenous regulation from host seeking to resting behavior is most pronounced 24 h post-blood meal [4, 10, 13, 24–28, 30, 31]. The differential expression of the associated molecular machinery may provide insights for further investigations into the link between gene expression and olfactory behavior [24].

In this study, we employ high throughput quantitative RNA sequencing to further the existing annotation of chemosensory genes and subsequently to investigate the influence of a blood meal on various chemosensory gene families in *C. quinquefasciatus* females, 24 h post-blood feeding, the quiescent period prior to engaging in pre-oviposition behavior. Key chemosensory genes within the olfactory apparatus are identified that are modulated between host seeking and blood fed females. The potential biological significance of this genetic regulation is discussed in the context of the behavioral switch between host seeking and resting in this anautogenous mosquito.

## Methods

### Rearing and tissue collection

*Culex quinquefasciatus* (Thai strain) were reared under normal laboratory culture conditions (27 ± 2 °C; 70 ± 2% relative humidity; 12 h:12 h light-dark photoperiod) [32]. Antennae from cold-anaesthetized reproductively mature adult females, 6 days post-emergence, were dissected directly into RNAlater® (Thermo Fisher Scientific, Sweden). To minimize the effect of the circadian rhythm associated with gene expression, the tissue was collected during the peak of host seeking in early scotophase, zeitgeber time (ZT) 15 ± 2 h [33], over multiple days. Collected tissue was stored at 8 °C overnight and then transferred to −80 °C until RNA extraction. While all mosquitoes were provided with *ad libitum* access to a 10% sucrose solution, only half of the adult females were provided with an opportunity to blood feed 24 ± 2 h prior to dissection. Females were offered sheep blood (Håtuna Lab, Bro, Sweden) from an artificial feeder (Hemotek Discovery Workshops, Accrington, UK). In total, 800 pairs of antennae were collected from females of each cohort, non-blood fed (nbf) and blood fed to completion (bf).

### RNA extraction and sequencing

Tissues were homogenized using a Vibra-Cell sonicator (VCX-130, Sonics and Materials, Newtown, CT) for 10 cycles at 70% amplitude, 1 s on and off pulses, repeated three times, interspersed with 30 s incubations on ice. Total RNA was extracted using an RNeasy Mini Kit (Qiagen, Sweden) according to the manufacturer’s protocol, including the on-column RNase-free DNase I treatment (Qiagen, Sweden). The RNA was quantified fluorometrically (Qubit, Life Technologies, Sweden) and then stored at −80 °C. An aliquot of RNA was shipped on dry ice to Eurofins Genomics (Munich, Germany) for library construction and single-end Illumina sequencing (3’ RNA-Seq) of the cDNA libraries (Illumina HiSeq 2500). Oligo-dT-based priming of total RNA to extract mRNA was used to generate cDNA libraries. Each library was divided into two technical replicates, which were run on separate channels.

### Read mapping and gene annotations

Prior to mapping, adapter sequences were removed from the raw reads and low quality bases (Phred score ≤20) from the start and end of each single read were clipped in a sliding window approach (window size 4 bp, minimum quality 20) using Trimmomatic 0.20 (http://www.usadellab.org). Reads shorter than a length threshold of 40 bp were also removed. Alignment of filtered reads to the reference-annotated genome forms the basis of RNA-Seq analysis [34]. CLC Genomics Workbench 9.0 (http://www.clcbio.com; Qiagen, DK) was used to map the sequenced reads to the reference genome in VectorBase (CPipJ2.2). While 95.6% of the total counted fragments mapped to the scaffolds from CPipJ2.2, the majority (55.1%) aligned to the predicted intergenic regions of the genome. This relatively low mapping frequency (40.5%) to the genic regions demonstrates that the latest genome annotation (CPipJ2.2) is still largely a prediction, which is unverified by transcriptome analysis, and is therefore unpredictable in both exon choice and transcript length. Hence, in this study the annotations for chemosensory genes were manually curated from the CPipJ2.2 supercontigs. Supported by the alignment of reads to scaffolds and sequence analyses, exon-intron boundaries and 3’ untranslated regions were modified for 158 chemosensory genes in total (Additional files 1, 2 and 3).

### RNA-Seq analysis

RNA-Seq analyses were performed using CLC Genomics Workbench 9.0 (https://www.qiagenbioinformatics.com/). Read count means, normalized between libraries, were chosen to quantify transcript abundance. The RNA-Seq libraries produced single-end reads from the 3’ end of each transcript present, providing a strong correlation between the read count and transcript abundance. A rigorous threshold of less than 10 mean counts across either replicate filtered out transcripts below a background level of abundance from the dataset, similar to the <1 RPKM filter that is commonly employed to reduce noise [24]. Genes with mean reads counts above this threshold were considered to be reliably expressed. Quantile-based normalization [35] was performed on each library to increase the detection sensitivity of differential expression for low abundance transcripts. The Kal’s weighted Z test [36], which relies on an approximation of the binomial distribution, was applied on the normalized read counts. It was controlled for false discovery rate (FDR) by applying the Benjamini-Hochberg correction [37]. This analysis generated weighted fold changes (FC) and FDR corrected *P* values that were used to detect differential expression. Genes that exhibited a FC > 2 and *P* < 0.05 were considered to demonstrate significant differential expression, while those with 1.5 ≥ FC ≥ 2 and *P* < 0.05 were considered not to be significantly different, but of potential interest. Normalized expression means were transformed with the addition of a constant (i.e. 1.1) to prevent values of infinitive fold change when one of the two libraries being compared exhibited no basal expression. For visualization, the mean counts were then log_2_ transformed to clearly distinguish genes of varying levels of abundance.

### Quantitative real time PCR

Quantitative real time polymerase chain reaction (qPCR) analysis was performed on select genes to verify the fold changes demonstrated by the transcriptome. Forward and reverse primer pairs (Additional file 4) were designed using Primer 3 (http://bioinfo.ut.ee/primer3-0.4.0/primer3/), with the following parameters: oligo size of 20-22 bp, melting temperature of ~60 °C, GC % content of 40–60%, 2 bp GC clamp at 3’ end, amplicon size between 80–150 bp, and preferably overlapping an intron/exon boundary. cDNA libraries for qPCR analyses were prepared from total RNA (extracted as above), based on Oligo(dT) priming using SuperScript III First-Strand Synthesis System for RT-PCR (Thermo Fischer Scientific, CA, USA), according to the manufacturer’s protocol. Four biological replicate cDNA libraries were generated for each bf and nbf cohort. The cDNA was stored at −20 °C until needed. Quantitative PCR was performed using the Platinum SYBR Green qPCR SuperMix-UDG w/ROX (Bio-Rad Laboratories, CA, United States), according to manufacturers’ protocol, with minor modifications for optimization. The reactions were carried out in 25 μl total volume, containing 12.5 μl of Bio-Rad Supermix, 0.5 μl of forward and reverse primers (Eurofins Genomics, Munich, Germany), 1 μl of cDNA and the remaining volume of DNAse-RNAse free, PCR-grade water. Amplification was performed on a BioRad CFX 96 (Bio-Rad Laboratories, CA, United States), with the following program: 2 min cycle at 50 °C, and 2 min at 95 °C, followed by 40 cycles of 30 s each at 95, 58 and 72 °C. Fluorescence readings were taken for each cycle during the elongation step at 72 °C. Melting curve analyses (65 °C to 94 °C in 0.5 °C steps) were performed after the 40^th^ cycle, to test the specificity of the product amplification. For each plate, a water control and negative cDNA synthesis control were included. Each primer pair was tested on three technical replicates for each biological replicate for both bf and nbf cohorts. Gene expression levels were determined using the ΔΔCq method [38]. Transcription levels per sample were normalized to a reference factor comprising the geometric means of the three most stable reference genes, *Elfa1* (CPIJ016188), *Orco* (CPIJ009573) and *RpS4* (CPIJ003013), and expressed relative to the mean of the control group (nbf) females. Gene expression levels were compared between genes per group (nbf and bf) using a two-tailed paired Student’s t-test following verification for normality and homogeneity of residuals using D’Agostino-Pearson test. Statistical significance was adjusted for multiple comparisons.

## Results and Discussion

### RNA sequencing data

Quantitative single-end sequencing of antennal mRNA from nbf and bf cohorts generated a combined sequencing depth of over 71 million cleaned reads (Additional file 5), which is similar to previous studies on related species that have produced total reads in the range of 65 to 240 million [24, 39–41]. Since the *C. quinquefasciatus* genome contains multiple gene family expansions, including olfactory and gustatory receptors [21], high sequencing depth generates sufficient coverage to detect transcripts with low abundance, *e.g.,* the chemoreceptor gene families [24, 42] that are the primary interest of this study. To validate the sufficiency of the coverage, the core eukaryotic genes mapping approach (Cegma) provides a set of reliable gene annotations, whose proportion in the genome assembly provides an estimate of the proportion of all known genes that are present [43]. Cegma genes are predominantly housekeeping genes that code for a group of highly conserved eukaryotic ubiquitous proteins [44]. Of the 358 Cegma genes (Additional file 6) identified in *C. quinquefasciatus*, transcripts for 331 were detected in the antennal libraries, indicating good sequence coverage of the transcriptome.

Quantile-based between-sample normalization was performed to correct for any read distribution bias amongst the libraries, thereby increasing the accuracy of quantitative comparisons among low expression transcripts [45]. This allowed for an increased sensitivity in detection of differentially expressed genes. The squared Pearson coefficient (r^2^) [46] of the between-replicate analyses for all four libraries approached one, implying close agreement between the technical replicates (Additional file 5). The increased coefficient following the normalization of the read counts reiterated the effectiveness of this normalization procedure.

### Overall expression profile

Single-end quantitative RNA-Seq detected a total of 15,187 transcripts in the antennal tissue of *C. quinquefasciatus* females. Out of these, 7,797 transcripts were reliably expressed in numbers above the rigorous cut-off used to control for background levels of abundance (Additional file 7). The majority of the Cegma genes were reliably expressed in the olfactory tissue, where, out of the 331 Cegma genes detected in *C. quinquefasciatus,* 290 were present above background abundance (Additional file 6).

**Fig. 1:**
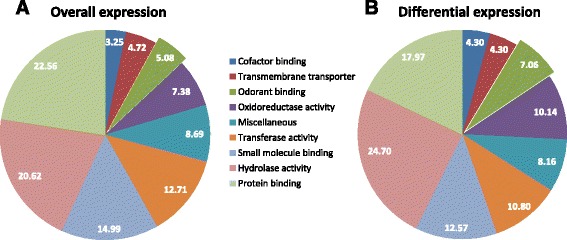
Level three gene ontology analysis of molecular functions in the antennae of female *Culex quinquefasciatus.* The total number of genes in the antennal transcriptome that are **a** reliably expressed overall and **b** differentially expressed between non-blood fed and blood fed cohorts

A level three gene ontology (GO) analysis of the molecular function was performed to gain an overview of the antennal gene expression, and to observe differences in the proportional representation of the genes involved in various molecular functions between transcripts reliably expressed in the olfactory tissue and transcripts differentially expressed between the nbf and bf cohorts (Fig. 1). Transcripts assigned ‘odorant-binding’ functions, and which include the majority of the chemosensory genes, represent 5.08% of the overall number of genes expressed in the antennae, and represent 7.06% of the genes which are differentially expressed in the nbf and bf antennae (Fig. 1), indicating that the regulation of genes with an odorant binding function are likely of importance in the behavioral switch between nbf and bf states.

Comparison between the transcriptomes of nbf and bf females yielded 714 genes that were significantly and differentially expressed (Kal’s *P* < 0.05 and FC > 2) in the antennal tissue (Additional file 7). The highest up- or down-regulation of gene expression post-blood feeding was observed for transcripts of various protein degrading enzymes (cathepsins), salivary proteins and yolk proteins (vitellogenins), similar to that observed in the *An. gambiae* antennal transcriptomes [24]*.* Two circadian genes, timeless and clock, were significantly down-regulated in the bf cohort, as was also observed in *An. gambiae* [24]. The majority of the reliably expressed Cegma genes (94.5%) in the olfactory tissue were not regulated in response to blood feeding, as expected (Additional file 6).

The highly expanded olfactory gene repertoire of *C. quinquefasciatus* [21, 40] is thought to reflect the opportunistic feeding behavior of *Culex* females on birds, humans and livestock, and the diversity observed in oviposition site choice [47]. Out of a repertoire of 180 putative *ORs* identified in *C. quinquefasciatus* [21], transcripts for 132 (73%) were detected in the antennae of 6 days post-emergence (dpe) females, consistent with a previous report [40], of which 96 *ORs* were reliably expressed above background abundance. This accords with the 56 (70%) *AgORs* and 66 (66%) *AaORs* detected in *An. gambiae* and *Ae. aegypti*, respectively [17, 24]. This expression correlates with the “one OR-to-one ORN class-to-one glomerulus” hypothesis when compared with the expected 50-60 glomeruli in the antennal lobe predicted by comparison with related *Culicine* species [12, 32, 48].

The IR gene family in *C. quinquefasciatus*, includes 73 members, of which transcripts for 62 *IRs* were detected in the antennae of 6 dpe females, consistent with a previous report [40], 30 of which were reliably expressed. This is in line with that reported for *An. gambiae* (23 out of 46) [19]. Expression for only one of the three co-receptors [49], *IR8a*, was detected above background abundance, as described previously [40]. Six members of the GR gene family were also expressed in the antennae of *C. quinquefasciatus*, similar in number to that in *An. gambiae* (13) and *Ae. aegypti* (6) [17, 24]. Both culicines show expression for the CO_2_-sensitive GR gene, *GR2*, as well as each expressing a single sugar-sensitive GR gene, *CqGR8* and *AaGr6* [24], whereas neither of these subfamily GR genes are shown to be expressed in the antennae of *An. gambiae* [24].

Out of a repertoire of 109 *OBPs* [50], transcripts for 74 *OBPs* were detected in the antennae, 63 of which were reliably expressed (58%), following a significant improvement in annotation compared to previous reports [40]. This is in line with that reported for *An. gambiae* (61%), while the expression of OBP genes in the antennae of *Ae. aegypti* (39%) is lower [17, 24]. The CSP gene family comprises 27 members in *C. quinquefasciatus* [40], out of which transcripts for 19 *CSPs* were detected in the antennae of females 6 dpe, with reliable expression for 12. In most mosquito species, the SNMP family comprises two members [51], *SNMP1* and *SNMP2*, whereas in *Culex*, the family has expanded to comprise four members, *SNMP1a, 1b, 1c* and *SNMP2* [40]. Reliable expression above background abundance was observed for only two *SNMPs* in the antennal tissue.

### Regulation of olfactory genes

For the majority of the chemosensory genes, an overall lower level of transcription was observed in the female antennae of bf (3.6 × 10^6^ reads) compared to nbf (3.8 × 10^6^ reads) mosquitoes. This differs from the change in overall level of transcription, which is found to be higher in the bf (14.7 × 10^6^ reads) compared to the nbf (12.8 × 10^6^ reads) mosquitoes (Additional file 7). This highlights the importance of the chemosensory function of the antennae during host seeking, and is in accordance with studies on *Ae. aegypti* and *An. gambiae* [17, 24]*.* For these species, a reduction in olfactory responsiveness towards host cues post-blood feeding has been well documented, behaviorally and physiologically [10, 13, 27, 28, 52]. Following a blood meal, the olfactory responsiveness of female mosquitoes to host cues gradually decreases until females are fully refractory to host odors, 24 h after a complete blood meal. By this time mosquitoes have located a resting site where females remain quiescent until the pre-oviposition behavior begins between 48 h and 96 h post-blood meal [28, 53].

*Aedes aegypti* females, 24 h post-blood feeding, are known to down-regulate their olfactory sensitivity towards lactic acid, a host cue [31], and up-regulate their sensitivity towards oviposition cues, such as indoles and phenols [13]. Similarly, in *An. gambiae*, down- and up-regulation of ORN sensitivity to host and oviposition cues, respectively, have been observed post-blood feeding [14], coinciding with the selective regulation of *OBPs*, *ORs* and *IRs* [24]. These changes are believed to modulate the chemosensory apparatus to inhibit the host-seeking response during the resting state and later to revive the system for subsequent gonotrophic events, including the pre-oviposition behavior [10, 13, 24]. It is noteworthy that the chemosensory system of *Ae. aegypti* begins to reconfigure its sensitivity towards oviposition site cues as early as 24 h post-blood meal [13].

#### Odorant receptors

**Fig. 2:**
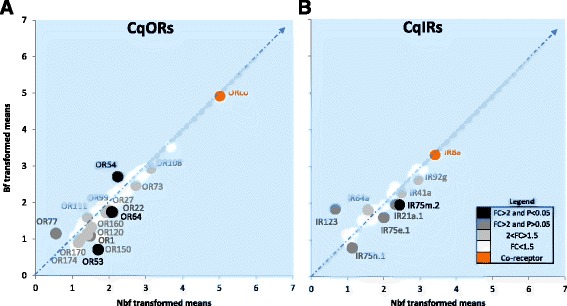
Differential regulation of chemosensory receptors in *Culex quinquefasciatus* antennae pre- and post-blood meal. The differential transcript abundance in the antennae of non-blood fed and blood fed females, 6 days post-emergence, are described for **a** odorant receptors (*ORs*) and **b** ionotropic receptors (*IRs*). Transcripts that exhibit significant differences in abundance (Kal’s test; *P* < 0.05), are denoted according to their weighted fold change (FC)

While the overall abundance of the tuning *ORs* in the antennae was reduced by one fifth in bf compared to nbf females, the obligate co-receptor, *ORco*, which had the highest transcript abundance in both cohorts, remained unregulated (Fig. 2a). This is in accordance with that of *AgORco* [24] and *AaORco* [17].The invariant transcript levels of mosquito *ORco* post-blood meal, as opposed to that of the obligate blood feeder *Rhodnius prolixus* [54], suggest different roles of the co-receptor in regulating the observed plasticity in olfactory responsiveness among hematophagous insects.

Changes in abundance were observed for the tuning *CqORs*, with 16 *CqORs* regulated in response to blood feeding (Fig. 2a). *CqORs* having higher transcript abundance in nbf females included *OR64* and *OR53* (listed in ascending magnitude of fold change) with FC > 2 and *P* < 0.05, and *OR22* and *OR1* with FC > 2 and *P* > 0.05. *CqORs* having higher transcript abundance in bf females included *OR54* with FC > 2 and *P* < 0.05, and *OR77* with FC > 2 and *P* > 0.05. Another ten CqORs of interest (1.5 < FC < 2) included *OR99, OR160, OR108, OR27, OR174, OR73, OR170, OR150, OR120* that had higher abundance in nbf females, and *OR111*, which had a higher abundance in bf females. The gene expression profiles of six of the differentially expressed OR genes determined by transcriptome analyses (*OR27, OR53, OR64, OR73, OR150,* and *OR170*), were confirmed using qPCR analyses (Additional file 8).

The ligand tuning of most CqORs has not been determined, and therefore a systematic effort to deorphanize these receptors will be required to address the functional relevance of the observed gene expression changes. Of the ORs present in higher abundance in the antennae of host seeking *C. quinquefasciatus* females (nbf), *OR73* is part of a *Culex*-specific expansion group [21]. *OR73* is tuned to multiple phenolic compounds including 4-methyl and 4-ethylphenol [55], which are components of human headspace collections [56, 57]. However, as of now there is no direct behavioral evidence documenting the use of phenols by *C. quinquefasciatus* females as host cues. Moreover, ethyl- and methylphenols are also known oviposition attractants [58]. Down-regulation of *OR73*, 24 h post-blood feeding, could thus be attributed to either the refractoriness to host cues or the delay of the onset of pre-oviposition behaviors. The down-regulated *OR1*, a receptor tuned to multiple host volatiles, including 1-hexanol, 1-octen-3-ol, 2-phenoxyethanol, and benzaldehyde [55], illustrates that ORs tuned to host volatiles are pivotal in regulating the behavioral switch from host seeking to resting.

#### Ionotropic receptors

The ionotropic co-receptor, *IR8a*, was the most abundant transcript, with invariant levels between the bf and nbf cohorts (Fig. 2b), consistent with that found in *An. gambiae* [24] and *Ae. aegypti* [17], but opposed to *R. prolixus* [54]. Thus, the regulation of chemosensory co-receptors, including *ORco*, in hematophagous insects seems to be a result of convergent evolution and diverse feeding strategies. In contrast, eight *CqIRs* in the antennal tissue were regulated in response to blood feeding (Fig. 2b). Six of these, *IR41a*, *IR92g, IR75h.1, IR21a.1, IR75e.1, and IR75m.2* were present in higher abundance in the nbf cohort, and two of them, *IR64a* and *IR123* were present in higher abundance in the bf cohort. The qPCR results (Additional file 8) demonstrated statistically significant differential regulation of six of the IR genes (*IR21a, IR64a, IR75e.1, IR75h.1, IR75m.2,* and *IR92g*), thus confirming the expression profiles described by the transcriptomic analyses. The functional characterization of IRs in mosquitoes is only now becoming available [19].

The conserved antennal *IR75* subfamily in *C. quinquefasciatus* comprises 15 members, of which 11 were reliably expressed, and three were down-regulated in response to blood feeding. IRs are involved in the detection of carboxylic acids and amines [10, 19, 59, 60] that are known host and oviposition volatiles, which have a low representation in the odor space of ORs [61, 62]. The *IR75* clade is suggested to respond to closely related volatiles [61, 63], and their selective down-regulation post-blood meal indicates their involvement in host selection. Interestingly, *IR123* and *IR64a* were upregulated 24 h post-blood feeding. In *Drosophila, IR64a* is responsible for acid avoidance responses [64], and the increased abundance of *IR64a* in the bf cohort may thus signal aversion to host carboxylic acids following a blood meal.

#### Odorant binding proteins

**Fig. 3:**
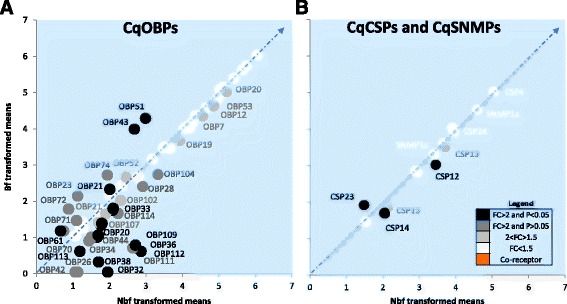
Differential regulation of other chemosensory proteins in *Culex quinquefasciatus* antennae pre- and post-blood meal. The differential transcript abundance in the antennae of non-blood fed and blood fed females, 6 days post-emergence, are described for **a** odorant binding proteins (*OBPs*) as well as **b** chemosensory proteins (*CSPs*) and sensory neuron membrane proteins (*SNMPs*). Transcripts that exhibit significant differences in abundance (Kal’s test; *P* < 0.05), are denoted according to their weighted fold change (FC)

Thirty-four *CqOBPs* in the antennae were regulated in response to blood feeding (Fig. 3a). Eight *CqOBPs* with FC > 2 and *P* < 0.05, eight *CqOBPs* with FC > 2 and *P* > 0.05, and another eight *CqOBPs* with 1.5 < FC > 2 were abundant in the nbf cohort. In contrast, four *CqOBPs* were abundant in the bf cohort with FC > 2 and *P* < 0.05, five *CqOBPs* with FC > 2 and *P* > 0.05 and one, *CqOBP52*, with FC of 1.67. Little information is available on the functional characterization of *CqOBPs,* without which few conclusions can be drawn from the expression data alone. Larger numbers of *CqOBPs* were up-regulated in bf females when compared to other chemosensory gene families. A similar trend was observed in *An. gambiae,* where most *AgOBPs* were down-regulated immediately after blood feeding and then up-regulated at 24 h post-blood meal [24]. This suggests that the olfactory apparatus of females is transiently changing at this early stage in the gonotrophic cycle, likely in response to the physiological and behavioral requirements.

#### Other chemosensory proteins

The highest transcript abundance among the *CSPs* was observed for *CSP4* and *CSP24*, neither of which were regulated in response to blood feeding. Five other *CSPs* were differentially expressed between the nbf and bf cohorts (Fig. 3b). Transcripts with higher abundance in nbf females included *CSP14* and *CSP12* with FC > 2 and *P* < 0.05, and *CSP15* and *CSP13* with 1.5 < FC < 2. *CSP23* was the only up-regulated *CSP* in the female antennae 24 h post - blood meal. Both of the expressed *SNMPs* were unregulated in response to blood feeding.

## Conclusions

In this study, we have demonstrated blood meal induced regulation of chemosensory genes in the antennae of *C. quinquefasciatus* females. We highlight candidate chemosensory genes that are differentially expressed between nbf and bf females. The functional characterization of these proteins can provide critical insights into the regulation of host seeking and post-blood feeding behaviors, and their de-orphanization can provide a basis for understanding odor coding in female *C. quinquefasciatus*. As such, this quantitative RNA-Seq analysis provides insights into the genetic regulation of the peripheral olfactory system of *C. quinquefasciatus* females.

## Additional files

Additional file 1: Lists the names and VectorBase IDs for *Culex quinquefasciatus* chemosensory gene families. (XLSX 22 kb)

Additional file 2: Lists the modified mRNA and peptide sequences of the *Culex quinquefasciatus* odorant receptor gene family. (XLSX 140 kb)

Additional file 3: *Culex quinquefasciatus* gene-set with corrected intron-exon boundaries and 3’ UTRs for 158 chemosensory genes. (TXT 25.6 mb)

Additional file 4: Lists the sequences, amplicon sizes, melting temperatures, GC % content and 3’ complementarity of the forward and reverse primer-pair for each of the reference genes and select *ORs* and *IRs* that were verified using qPCR analyses. (DOCX 29 kb)

Additional file 5: Describes the sequencing depth and Pearson coefficients of the technical replicates from non-blood fed and blood fed libraries. (XLSX 10 kb)

Additional file 6: Lists the names, read count means and Kal’s test statistics for the Cegma genes identified in *Culex quinquefasciatus*. (XLSX 100 kb)

Additional file 7: Lists the read count means and Kal’s test statistics for all transcripts known to be reliably expressed (present above the cut-off filter). (XLSX 1909 kb)

Additional file 8: Depicts relative gene expression of select *IRs* and *ORs* in non-blood fed and blood fed libraries, as determined by real time quantitative polymerase chain reaction. (PDF 86 kb)

## Abbreviations

bf: Blood fed
bp: base pair
cDNA: complementary deoxyribonucleic acid
Cegma: Common eukaryotic gene mapping approach
CSP: Chemosensory protein
FC: Weighted fold change
FDR: False discovery rate
GC: Guanine and cytosine
GO: Gene ontology
GR: Gustatory receptor
IR: Ionotropic receptor gene
nbf: non-blood fed
OBP: Odorant-binding protein
OR: Odorant receptor
ORco: Obligate OR co-receptor
ORN: Olfactory receptor neuron
qPCR: quantitative real-time polymerase chain reaction
RNA: Ribonucleic acid
RNA-seq: Quantitative mRNA sequencing
RPKM: Reads per kilobase of transcript length per million mapped reads
SNMP: Sensory neuron membrane protein
