# Supplementary material for: Blood meal induced regulation of the chemosensory gene repertoire in the southern house mosquito
Source: BMC Genomics. 2017 May 19;18:393. doi: 10.1186/s12864-017-3779-2 (PMC5437716; doi:10.1186/s12864-017-3779-2)
Supplement: Supplementary file 4 — Lists the sequences, amplicon sizes, melting temperatures, GC % content and 3’ complementarity of the forward and reverse primer-pair for each of the reference genes and select ORs and IRs that were verified using qPCR analyses. (DOCX 29 kb) [file 12864_2017_3779_MOESM4_ESM.docx]

OLIGO [start](http://bioinfo.ut.ee/primer3-0.4.0/primer3_www_results_help.html#PRIMER_START)  [len](http://bioinfo.ut.ee/primer3-0.4.0/primer3_www_results_help.html#PRIMER_LEN)  [tm](http://bioinfo.ut.ee/primer3-0.4.0/primer3_www_results_help.html#PRIMER_TM)  [gc%](http://bioinfo.ut.ee/primer3-0.4.0/primer3_www_results_help.html#PRIMER_GC)  [any](http://bioinfo.ut.ee/primer3-0.4.0/primer3_www_results_help.html#PRIMER_ANY)  [3'](http://bioinfo.ut.ee/primer3-0.4.0/primer3_www_results_help.html#PRIMER_REPEAT) [seq](http://bioinfo.ut.ee/primer3-0.4.0/primer3_www_results_help.html#PRIMER_OLIGO_SEQ)

Or170

LEFT PRIMER 6 20 60.50 55.00 2.00 2.00 CTGGAGTGTGCTGTTGTTCG

RIGHT PRIMER 112 20 60.02 50.00 4.00 2.00 CGGAAGTCAAAAAGCTACGC

PRODUCT SIZE: 107, PAIR ANY COMPL: 4.00, PAIR 3' COMPL: 2.00

Ir75h.1

LEFT PRIMER 2968 20 60.28 50.00 5.00 1.00 ATGACGCTTAGCAACGAACC

RIGHT PRIMER 3069 20 59.79 50.00 3.00 2.00 AAACACGCACACTGCTATGG

PRODUCT SIZE: 102, PAIR ANY COMPL: 5.00, PAIR 3' COMPL: 2.00

RefRps4

LEFT PRIMER 397 19 60.07 52.63 4.00 0.00 AAGAAGGTGCCGTTCATCC

RIGHT PRIMER 549 20 60.09 50.00 6.00 2.00 GGTGATCATGCACAGATTGC

PRODUCT SIZE: 153, PAIR ANY COMPL: 4.00, PAIR 3' COMPL: 1.00

RefElf1a

LEFT PRIMER 3 20 60.03 50.00 2.00 0.00 GCCGAAGAATAAGGGAAAGG

RIGHT PRIMER 130 20 61.01 45.00 3.00 1.00 TTCCGAGCATTTTTGTGACC

PRODUCT SIZE: 128, PAIR ANY COMPL: 4.00, PAIR 3' COMPL: 2.00

Orco

LEFT PRIMER 1190 20 59.84 55.00 3.00 2.00 CCCAGGTCTTTCTGTTCTGC

RIGHT PRIMER 1314 20 59.53 50.00 4.00 0.00 GATCTGGACGAAGGTTTTGG

PRODUCT SIZE: 125, PAIR ANY COMPL: 5.00, PAIR 3' COMPL: 2.00

Or150

LEFT PRIMER 539 20 59.90 50.00 3.00 2.00 AAATCACGCACTCTGACACG

RIGHT PRIMER 683 20 59.14 50.00 6.00 2.00 GTGTCGATTTGTCCACAAGC

PRODUCT SIZE: 145, PAIR ANY COMPL: 5.00, PAIR 3' COMPL: 1.00

Or27

LEFT PRIMER 141 20 60.17 50.00 6.00 1.00 GGTGATCATGTTCCCAAAGG

RIGHT PRIMER 270 20 60.63 50.00 3.00 3.00 CCTCTTCAGTGCCAAAATGG

PRODUCT SIZE: 130, PAIR ANY COMPL: 4.00, PAIR 3' COMPL: 1.00

Or53

LEFT PRIMER 677 20 59.98 50.00 4.00 2.00 TTGACCTGCTGAAGATGTCG

RIGHT PRIMER 820 20 59.47 55.00 4.00 3.00 CACGATAGACCAGCTCAACG

PRODUCT SIZE: 144, PAIR ANY COMPL: 6.00, PAIR 3' COMPL: 2.00

Or64

LEFT PRIMER 355 20 59.84 55.00 6.00 2.00 CTGTTCCAGGAGGAAACAGC

RIGHT PRIMER 483 20 59.61 50.00 6.00 2.00 TTTGTGTCCCGACAGTAACG

PRODUCT SIZE: 129, PAIR ANY COMPL: 5.00, PAIR 3' COMPL: 1.00

Or73

LEFT PRIMER 1 20 59.79 50.00 3.00 0.00 ATGTCGTCCATCAACCTTCC

RIGHT PRIMER 110 20 59.51 55.00 6.00 2.00 GTCCTCGGGTGATATCTTGC

PRODUCT SIZE: 110, PAIR ANY COMPL: 5.00, PAIR 3' COMPL: 0.00

Ir21a.1

LEFT PRIMER 798 20 59.47 50.00 4.00 2.00 GAAGTTTTCCCGTACCAACG

RIGHT PRIMER 899 20 59.97 50.00 3.00 2.00 ACAAATGGAGGTTGGTGAGC

PRODUCT SIZE: 102, PAIR ANY COMPL: 6.00, PAIR 3' COMPL: 3.00

Ir64a

LEFT PRIMER 1682 20 60.21 45.00 6.00 2.00 TGGAAATTTGCGAGCTTACC

RIGHT PRIMER 1781 20 59.13 55.00 5.00 2.00 GTTATGACATCCCGGTAGGG

PRODUCT SIZE: 100, PAIR ANY COMPL: 4.00, PAIR 3' COMPL: 3.00

Ir75e.1

LEFT PRIMER 337 20 60.54 55.00 4.00 1.00 CCAAGTACGAACACCGAAGG

RIGHT PRIMER 465 20 59.95 60.00 4.00 1.00 GTCTCCAGCGATCTGACTCC

PRODUCT SIZE: 129, PAIR ANY COMPL: 4.00, PAIR 3' COMPL: 2.00

Ir75m.2

LEFT PRIMER 344 20 59.71 50.00 6.00 2.00 GTTCGGTTTCAGTTCGAAGG

RIGHT PRIMER 451 20 60.58 45.00 4.00 2.00 AAATGCGATTGTGTGTGTCG

PRODUCT SIZE: 108, PAIR ANY COMPL: 3.00, PAIR 3' COMPL: 0.00

Ir92g

LEFT PRIMER 385 20 60.07 50.00 5.00 0.00 ATCGACGAGAATGGAACTGG

RIGHT PRIMER 487 20 60.32 50.00 6.00 2.00 GAATGTCTTGGACCCAATCG

PRODUCT SIZE: 103, PAIR ANY COMPL: 4.00, PAIR 3' COMPL: 1.00
