# Supplementary material for: Blood meal induced regulation of the chemosensory gene repertoire in the southern house mosquito
Source: BMC Genomics. 2017 May 19;18:393. doi: 10.1186/s12864-017-3779-2 (PMC5437716; doi:10.1186/s12864-017-3779-2)
Supplement: Supplementary file 8 — Depicts relative gene expression of select IRs and ORs in non-blood fed and blood fed libraries, as determined by real time quantitative polymerase chain reaction. (PDF 86 kb) [file 12864_2017_3779_MOESM8_ESM.pdf]

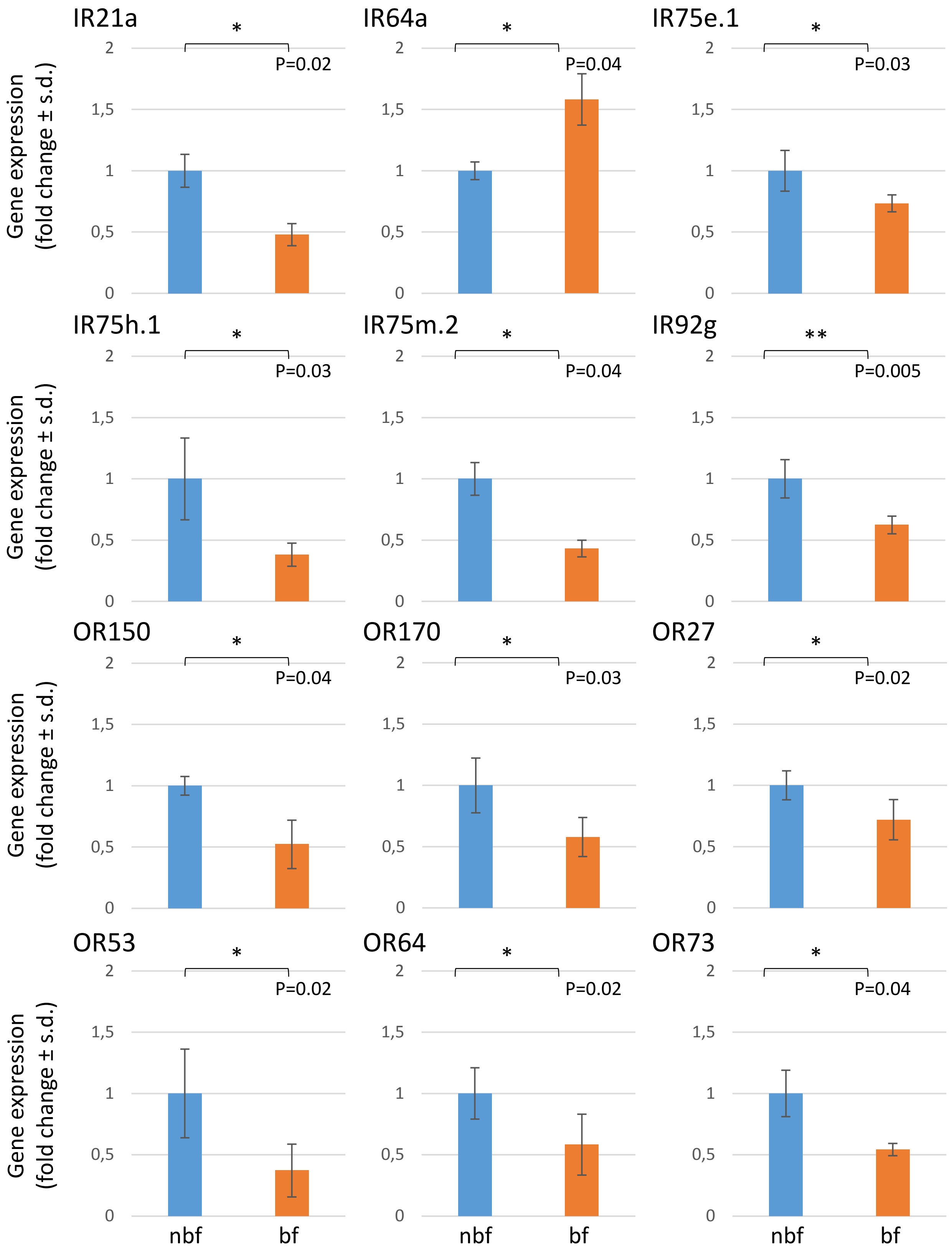

Additional figure 8: Relative gene expression of select ionotropic receptors (IRs) and odorant receptors (ORs) in host seeking (nbf) and 24 h post-blood fed (bf) *Aedes aegypti* females (6 days post-eclosion), as determined by real time quantitative polymerase chain reaction (mean fold change ± standard deviation), reflects the pattern and significance of the receptor gene expression as observed in the transcriptome analyses. Significant change in relative gene expression levels are denoted by asterisks (two-tailed paired Student's t-test, *P<0.05, **P<0.01).
